# Domesticating models: On the contingency of Covid-19 modelling in UK media and policy

**DOI:** 10.1177/03063127221126166

**Published:** 2022-10-13

**Authors:** Lukas Engelmann, Catherine M Montgomery, Steve Sturdy, Cristina Moreno Lozano

**Affiliations:** University of Edinburgh, Edinburgh, UK

**Keywords:** Covid-19, modelling, performativity, pandemic policy, media analysis

## Abstract

Our article traces the representation of pandemic modelling in UK print media from the emergence of Covid-19 to the early stages of implementing the first UK-wide lockdown in late March 2020. Covid modelling, it is widely assumed, has shaped policy decisions and public responses to the pandemic in unprecedented ways. We analyse how the UK print media has configured modelling as a significant evidence tool in the representation of the pandemic. Interrogating assumptions about infectious disease modelling, we ask why models became the trusted tool of choice for knowing and responding to the Covid pandemic in the UK. Our analysis has yielded four different periods in the evolution of intersecting policy and media frames. Initially, modellers, policymakers and media alike emphasized uncertainty about available data, and hence the speculative character of modelled projections, thus justifying a ‘wait and see’ approach to government intervention. With growing public pressure for government action, policy and media frames were adjusted to emphasize the importance of timing interventions for best effect, with modelling evidence mobilized to justify inaction. This gave way to a period of crisis, as the press increasingly questioned the reliability of the existing models and policies, leading modellers and policy makers to dramatically revise their projections. Finally, with the imposition of the first UK lockdown, policy and media frames were brought back into alignment with one another, in a process of domestication through which the language of modelling became a basic resource for the discussion of the epidemic. Our epistemological microhistory thus challenges general accounts of the impacts of pandemic modelling and instead emphasizes contingency and interpretative flexibility.

Modelling has been a prominent feature of the UK’s public and political discourse on Covid. Over the first few weeks of the emerging pandemic, modelling swiftly became synonymous with the science that politicians professed to follow, and folk epidemiologists flocked to social media to debate how to #flattenthecurve (Montgomery & Engelmann, 2020). Models produced by UK-based academics, made available to the government via its Scientific Advisory Group on Emergencies (SAGE), were mobilized by policy makers to explain and justify their responses to the developing crisis. The UK print media has reported on modelling from the earliest reports on the emerging epidemic in Wuhan to the UK-wide lockdown in March 2020 and across the pandemic’s first waves. Despite remarkable discontinuities in the recommendations made, and notwithstanding a significant crisis of credibility, models had become domesticated in UK print media as pandemic vernacular. Modelling is widely perceived as a leading form of expertise in the UK’s domestic response to the pandemic.

The epistemic character of predictive models, and their role in policymaking, has long been a source of interest for social scientists. Within science, models play an ambiguous, often intermediary role between the theoretical and experimental, abstract and concrete – and between science and policy application (Sismondo, 1999). One line of research into models and policy – much of it relating to climate modelling – has focused on this ambiguity, stressing the uncertainties and accommodations inherent in model-based predictions and in the back-and-forth between models and policy decisions. Such uncertainties and contingencies are often glossed over to invest policy with authority. Authors including Wynne and Shackley have called for them to be acknowledged so that the varied determinants of policy-making and modelling may be taken into account (Shackley et al., 1998; Wynne, 1984, 2010). Noting the role of ‘hubristic’ reliance on models that led to the economic crash of 2008, Saltelli and colleagues argue for greater transparency in representing the shared normative values or frames that structure the science-policy nexus, so that the specific historical and social conditions that inform model-based decisions remain open to scrutiny (Saltelli & Funtowicz, 2014; Saltelli & Giampietro, 2017).

Similar observations have been made of infectious disease modelling. Leach and Scoones (2013) have shown that Ebola and H5N1 influenza models have ‘social and political lives’ that encompass ‘the social, cultural and political norms and values that shape the development of particular models, and which they carry and project’; models are not epistemically neutral, but are ‘co-constructed with particular policy narratives about the disease problem’ (Leach & Scoones, 2013, pp. 10, 15). Leach and Scoones see this as reason to seek plurality in modelling and in policy advice. Law goes further, arguing that in the case of foot and mouth disease, UK policy makers opted for an ‘unnecessarily and inappropriately draconian’ cull precisely because the model on which it was based was ‘technically, politically, socially, and organizationally opaque’. Law follows Santelli and colleagues in calling for ‘political technologies that are, by contrast, relatively transparent, and therefore contestable’ (Law, 2008).

On this view, models’ value to policymakers lies as much in their ability to obscure political judgement behind a veneer of technical reason as in their ability to predict or prescribe what might or should happen; like other ‘evidence tools’ adduced to support policy, models’ ‘utility lies primarily in their symbolic value as markers of good decision making’ (Smith & Stewart, 2015).

But models do more than just gloss political decisions. Enrolled into policy through ‘assemblages of implementation’ and evidence-making, models not only ‘shap[e] rapid policy responses in infection control’; they also ‘entangle in social life as public concerns’ (Rhodes & Lancaster, 2020, pp. 177–178). Anderson concurs, charting a thirty-year history of modelling epidemic response in the UK to stress how Covid modelling has served to delimit the possibilities of intervention and reaction by ‘enacting a performance of calculation and control’ (Anderson, 2021, p. 177). Epidemic modelling has not just informed crucial government decisions but has assumed a performative quality in its capacity to shape the pandemic reality that we currently inhabit. Such studies have done much to elucidate how mathematical models acquire agency in policy, both in relation to Covid and more generally. But the case of Covid also provides an opportunity to ask pointed questions about the role models play in relation to particular policy decisions.

Over the course of just a week in mid-March 2020, the UK government dramatically changed its policy on Covid, from one of minimal, narrowly targeted interventions, to the imposition of a ‘lockdown’ – a sweeping combination of social distancing and other measures that massively restricted large areas of social and economic activity. As policy decisions go, this one was remarkable both for the speed with which it was enacted and the extent of its impact on social life. It was also notable for the prominent involvement of modellers, visible far beyond the confines of the science and policy community. In particular, a paper published by Neil Ferguson on 16 March 2020 has been widely held to have marked a turning point in the government’s response to the pandemic.

Two recent papers have looked in some detail at how models were implicated in this policy shift, and what role they played in the process of decision-making. Evans (2022) frames his analysis with a counterfactual. With the benefit of hindsight, it is thought that lockdown should have been imposed much sooner. Why did policymakers not act in a more timely fashion? Based on close reading of SAGE minutes and other policy documents, Evans argues that ‘both the government and the members of SAGE appear to have set the bar for “usable” knowledge at a very high level and that this delayed the implementation of policies to contain the pandemic’ (Evans, 2022, p. 54). As Evans sees it, policymakers and their scientific advisors deferred action until the pandemic could be modelled with a degree of certainty that might have been appropriate for scientific knowledge production, but went well beyond what was necessary to motivate precautionary measures.

Rhodes and Lancaster (2022) adopt a similar line, though framing it more positively, when they seek to understand ‘how mathematical models are made “evidence enough” and “useful for policy”’. They also cast their net more widely than Evans, using interviews to elicit modellers’ retrospective accounts of the events of March 2020, as well as policy documents from the time. They show that, alongside the kind of ‘data story’ that Evans narrates, these sources can be used to construct a parallel ‘assemblage story’ in which the impetus to action came from the entanglement of multiple actors (Rhodes & Lancaster, 2022, p. 5). Policy decisions were driven by the complex dynamics – affective as much as epistemic – that animated this assemblage. Rhodes and Lancaster conclude: ‘The evidence that models make, and how models are constituted as useful in policy, is a matter of situational performance, wherein models are brought to life as fluid elements of their implementation events’ (Rhodes & Lancaster, 2022, p. 8).

Both articles, it should be noted, seek primarily to determine what kinds of conditions were sufficient to precipitate policy action. Neither looks in any detail at how and why policy makers or their scientific advisors decided what form that action should take. If anything, both Evans and Rhodes and Lancaster take for granted that action meant lockdown – a consequence, perhaps, of the strong element of hindsight that informs both inquiries. The narratives they recount are consequently progressive, even teleological. For Evans, the story is one of accumulating evidence and certainty; for Rhodes and Lancaster, it is actors and affective urgency that accumulate within the science-policy assemblage. For both, the end point of accumulation is the decision to impose lockdown.

We offer a different kind of story – one that gives much greater prominence to contingency and discontinuity in policy, and that casts a different light on the agency attributed to models. Crucially, it focuses not just on the narrow science-policy nexus on which Evans and Rhodes and Lancaster focus, but brings in the press and the views that were expressed there concerning both government policy and the advice offered by modellers. Specifically, we use media analysis to show how and under what circumstances modelling became ‘the story’ of the Covid pandemic in the UK print media and how, in turn, the UK print media framed models as guiding the UK’s response to the pandemic.

As is widely recognized, mass media not only reflect the meaning of events in the world but are active participants in their production (Boero, 2013; Henderson & Hilton, 2018; Lupton, 1999). Henderson and Hilton note that ’media reporting both reflects and shapes cultural ideas about public health issues and, perhaps more importantly, frames solutions and responsibilities in ways which are politically charged and have policy consequences’ (Henderson & Hilton, 2018, p. 374). In our analysis, we combine media analysis with close reading of relevant policy documents, such as reports from SAGE meetings, and policy events including key announcements, to elucidate the complex interactions between science, policy, and the media through which models framed and were framed, configured and were configured, for public health (Lynch et al., 1990; Pickersgill et al., 2017).

Our media analysis focuses exclusively on print media in the UK, in light of the locally specific expectations, constraints and politics of presentation characteristic of these news outlets (Altheide & Schneider, 2013). We exclude social media from our analysis. While social media were undoubtedly important in reflecting and producing public sentiment throughout the pandemic, our aims were adequately served by concentrating on the more circumscribed world of print media and professional journalism, with its close and longstanding relationship with policy. We used Lexus-Nexis to search for articles that consider both Covid and modelling, with keyword searches including variations of these search terms, such as coronavirus or SARS-CoV-2, and modellers, models or modelling. The search spanned from the 1st of January 2020 to the 31st of March 2020 and focused on a broad spread of newspapers from the UK, including both broadsheets and tabloids across the political spectrum. Resulting articles were collected from the *Daily Mail*, the *Daily Record*, the *Guardian*, the *Herald*, the *Mirror*, the *Sun*, the *Daily Telegraph* and the *Times*. The initial search resulted in 3,687 articles. After scanning the articles and removing duplicates and irrelevant meanings of models and modelling, we arrived at a sample of 111 articles for analysis (see Supplemental Appendix 1). Since Lexus-Nexis coverage is not exhaustive, we augmented our search by looking for publications in the same newspapers by journalists whom we identified as taking an interest in Covid modelling. Articles were considered to speak directly to our research questions if they committed significant proportions of the text to explaining or interpreting pandemic modelling, if they represented policy decisions as due to or supported by such models, if they commented on the role of models or modellers in the policy process, or if they anchored their own inferences in evidence provided by modelling.

Our analysis of these sources drew on frame analysis (Goffman, 1974; Rein & Schön, 1996) to investigate how the media characterized the problem posed by Covid on the one hand, and the role of modelling in proposing responses to that problem on the other. This enabled us to identify the framings, in the sense of particular configurations of problem-plus-response, that predominated within the media at particular times, as well as what counter-framings were articulated, and how these framings and counter-framings changed and shifted over time. We distinguished this ‘media frame’ from what we call the ‘policy frame,’ by which we mean the way that government policy makers, including the modelers who advised them, used models to characterize the Covid pandemic and how to respond to it. By following not just the different framings of Covid that dominated the media and policy frames over time, but also the different degrees of alignment and misalignment between those frames, we were able to characterize four consecutive periods in the relationship between modelling and Covid policy. As set out in the following section, these were: (i) an initial period of speculation in which models were seen to have limited impact on policy, (ii) a period of prevarication during which models helped to justify delay in the introduction of preventive measures, (iii) a period in which both policy and modelling underwent a substantial crisis of credibility, and in which both were radically reoriented, and (iv) a period of domestication in which the press gave its assent to models, while retrospectively constructing an account of modelling as an essential guide to policy.

A key finding to emerge from this analysis is that modelling did not appear as a unified or consistent body of scientific method or evidence, within either the policy frame or the media frame. On the contrary, it incorporated multiple models and points of view and was marked by striking shifts in perspective and assumptions. Modellers close to policymakers clearly observed ‘the rules of the game’ that largely aligned with and supported policy decisions, as Cairney (2021) argues. But our analysis shows that the game itself changed dramatically over time: Across the four periods that we identify, modellers themselves engaged with and adapted to shifting sentiments in the political and the public sphere, demonstrating substantial ‘interpretive flexibility’ in their science (Star, 2010). It was this adaptability, above all, which made it possible for models to be accepted as authoritative statements on the state and future of the pandemic (Christley et al., 2013; Sismondo, 1999), and thereby to create rather than describe the pandemic as an object of political and public knowledge.

Our article therefore emphasizes the importance of temporal analysis as a means of demonstrating the processes by which models acquired authority over time, not just in policy circles, but in the print media. And by paying attention to discontinuity as much as the accumulation of evidence or the elaboration of an assemblage, it highlights the importance of political and scientific contingency. The models’ apparent capacity to define and to determine the shape of the pandemic, we argue, was thus the outcome of a temporally contingent process of domestication. Moreover, that domestication involved a retrospective projection of a continuous authority of modelling which effectively obscured the very discontinuities and contingencies that made it possible.

## Analysis: From speculation to domestication

### Speculation: January 1^st^–February 28^th^

Through January and February 2020, the UK press reported the emergence of what would come to be known as the Covid pandemic, from the first outbreak and dramatic lockdown in Wuhan, China, to the first imported cases in the UK in late January, to the local lockdown imposed on small areas of northern Italy towards the end of February. From the start, this reporting was coloured by speculation about the future: Could the disease become established in the UK and what might be the consequences? To inform their speculations, reporters drew on earlier epidemics such as SARS and Ebola, as well as the influenza pandemics of 1918 and 2009. They also included the work of infectious disease modellers who were at the time endeavouring to determine the epidemiological characteristics of Covid and predict its likely trajectory.

The UK press had long been acquainted with the work of infectious disease modellers, who had played a highly visible role in British policy responses to epidemics since the AIDS crisis of the 1980s (Mansnerus, 2015), and who were now recruited to the Government’s contingency planning for a possible Covid outbreak in the UK. In 2009, the UK Government had set up the Scientific Pandemic Influenza Modelling Group (SPI-M) to provide advice on influenza preparedness. Two of SPI-M’s most prominent members – Neil Ferguson of Imperial College London (ICL) and John Edmunds of the London School of Hygiene and Tropical Medicine (LSHTM) – were among the attendees when the Government convened its Scientific Advisory Group for Emergencies (SAGE) for a ‘precautionary’ review of the ‘Wuhan coronavirus’ on 22 January 2020 (SAGE, 2020a). Six days later, SPI-M in its entirety was incorporated as ‘a formal sub-group of SAGE for the duration of this outbreak’ (SAGE, 2020b). From that point on, SPI-M would provide regular input into SAGE’s deliberations and advice to government.

SAGE met behind closed doors, and minutes and other documents were not at that time released to the press or the public. Elements of the SPI-M findings were selectively shared through formal and informal press briefings, while modellers themselves also spoke directly with reporters. Consequently, reports of modellers’ projections of the possible consequence of Covid for the UK began to feature increasingly frequently, particularly in the broadsheet press. It is notable that, during this period, the press broadly reflected the same concerns and calculations that drove the modellers’ input into SAGE.

Specifically, SPI-M was tasked with helping SAGE to prepare for the possibility of a serious outbreak in the UK by advising on ‘actions the UK could take to slow down the spread of the outbreak domestically’ (SAGE, 2020b). Before they could offer such advice, however, the modellers needed to consider just how serious such an outbreak might be. Much of February was devoted to drawing up and refining a ‘Reasonable Worst Case’ (RWC) scenario for SAGE to work with. Initially, the modellers had very little data to inform their RWC. The first SAGE meeting of 22 January had noted the committee’s doubts about the quality of the data coming out of China and the uncertainty surrounding case numbers, incubation period, reproduction rate and other factors (SAGE, 2020a). Consequently, the modellers began by basing their projections on a SPI-M model for pandemic influenza from 2013 and updated in 2018 (SAGE, 2020b). Those projections were reviewed and updated regularly as new data on Covid became available, and by 26 February, estimates for most of the key epidemiological parameters that were expected to determine the size and shape of an outbreak had been incorporated into the models (UK Government, 2020a).

Press reporting followed a similar trajectory. Modellers and their work on Covid first appeared in *The Guardian* on 17 January – five days before the first SAGE meeting – following the release of a report by Ferguson’s group at Imperial College earlier that day (Imai et al., 2020). The report sought to extrapolate from the detection of three cases of Covid outside China – the first such cases to be identified – to estimate the likely total number of cases that had so far occurred within China. *The Guardian* quoted the report’s conclusion that there could be ‘anywhere from 190 to over 4,000’ such cases (GU001).^1^ On the same day, *The Guardian*’s health editor Sarah Boseley wrote in another article that Ferguson had already increased his upper estimate to possibly as high as 9,700, based on the mismatch between official counts of cases and reports of overwhelmed hospitals in Wuhan (GU002). Four days later, Boseley reported that Ferguson had further increased his estimate of the total number of cases based on his assumption of a reproductive rate of 2.5 to 3: ‘My best guess now is perhaps 100,000 cases right now’, he told Boseley (GU003).

From reporting modellers’ estimates of how many people were already infected, press discussion soon moved on to estimates of mortality rates. In that respect, the suggestion that case numbers were being systematically under-reported provided grounds for a degree of reassurance. Writing in *The Telegraph* on 10 February, global health security correspondent Anne Gulland questioned reports that had been circulating of a 20% mortality rate for Covid cases. This figure was hugely inflated, she suggested, ‘because only the most severe cases of the disease in China are being tested’. According to Ferguson, she noted, a similar failure to record cases with mild symptoms had characterized ‘the influenza pandemics of the 20th century’, leading to under-estimates of the true infection rate. If that was true of Covid too, she observed, it would likely indicate a much lower mortality rate than reported (TE001). She reiterated that view a week later: While concern was mounting that Covid might now be spreading undetected outside China, ‘mathematical modellers at Imperial College, London believe that only one in 10 cases of the disease are being recorded in China and the true death is likely to be much lower’ (TE002).

As more data became available and the SAGE modellers began to refine their Reasonable Worst Case, press interest turned increasingly towards what might happen if a Covid outbreak were to occur in the UK. In *The Times*, health editor Chris Smyth explicitly considered the RWC on 15 February. Smyth’s message was strikingly ambivalent. On the one hand, he observed, the RWC ‘anticipates hundreds of thousands of deaths and intensive care units forced to make “hard choices” about prioritising people’. On the other hand, he argued, it suggested that the death rate would be lower than many feared, as ‘even in the worst pandemic only 1 per cent to 4 per cent of those infected would need hospital treatment’ (TI001). The same ambiguous diptych appeared two days later in a *Times* article by Fariha Karim and Didi Tang, who reported LSHTM modeller John Edmunds as saying that ‘the government was working to a theory that as many as 33 million people could be infected in the UK, although not all of them would become seriously ill’ (TI002).

Reading these reports with the wisdom of hindsight, what stands out is the insouciance with which the press spoke of figures that now appear quite horrifying. By late February, SPI-M was calculating that, in a RWC, as many as 80% of Britons could become infected, of whom 1% might die, resulting in an estimated 520,000 ‘excess deaths’.^2^ Writing in August 2022, after the UK has experienced the collective trauma of nearly 187,000 deaths from Covid, these projections appear shocking. Yet they were repeatedly reported in the press, often without further comment.

This tells us much about how the press framed modellers’ accounts of Covid at that time. Despite the arrival of the first identified cases in the UK, the epidemic was still presented as something that was happening elsewhere and that was difficult to characterize with any degree of certainty. Modellers were portrayed as struggling to get a clear picture of how the disease behaved, while any conclusions they drew from their models were framed as inherently uncertain. Towards the end of January, for instance, an editorial in *The Guardian* emphasized the compromised quality of available data and called for a cautiously critical approach to evaluating the developing picture of the epidemic (GU004, GU006). This was echoed, as seen above, in commentaries in *The Times* and *The Telegraph* on the difficulty of estimating actual infection and mortality rates.

Throughout this period, then, modelling was framed primarily as a way of speculating about the nature and possible future of Covid. It provided a way of interrogating and making sense of unreliable information from distant sources. But it was itself uncertain, while the lessons it was seen to offer were as often reassuring – correcting overly pessimistic estimates of mortality rates, for instance – as they were disturbing. As for the Realistic Worst Case, it was presented as just that: not as a prediction of what *would* happen, but as a precautionary assessment of what *could* happen if the worst came to the worst. The huge numbers of deaths it posited were no more than a theoretical upper bounds. Indeed, it was not even clear whether the epidemic would ever take hold in the UK, never mind how severe it might be. Talk of the modellers’ RWC was thus cast firmly in the subjunctive mood. It was this that enabled the press to talk of hundreds of thousands of deaths with little apparent sign of alarm.

### Prevarication: 1st−12th March

Whereas the first phase of UK press coverage of Covid modelling was coloured by speculation about *whether* the disease could become established in the UK, the first two weeks of March saw a shift to talking about *when*. The shift was prompted not just by reports of the rapid progress of the disease in Italy and other countries, but also by a growing conviction that it was only a matter of time before it took root in the UK. By 3 March, SAGE was discussing a SPI-M consensus statement that noted that ‘It is highly likely that there is sustained transmission of Covid in the UK at present. It is almost certain that there will be sustained transmission in the UK in the coming weeks’ (SPI-M-O, 2020). This statement led to a ramping up of the UK government’s policy activities. On 1 March, the Civil Contingencies Committee (COBR), a standing committee that can be activated to address matters of national emergency or disruption, was for the first time convened to consider Covid. Two days later, on 3 March, the Department of Health published its first Coronavirus Action Plan. UK policy had so far focused primarily on containing the disease through measures to identify and isolate cases as they arrived in the country, the Action Plan stated. Now, however, ‘Our experts are considering what other actions will be most effective in slowing the spread of the virus in the UK, as more information about it emerges’. Measures under consideration included ‘population distancing strategies (such as school closures, encouraging greater home working, reducing the number of large-scale gatherings) to slow the spread of the disease throughout the population’ (UK Government, 2020b).

At the same time, however, the Government signalled its intention to put off introducing such measures until such time as it judged the benefits to outweigh the costs. As the Action Plan put it: ‘We will aim to minimise the social and economic impact, subject to keeping people safe. Such judgements will be informed based on the best available and most up-to-date scientific evidence, and take into account the trade-offs involved’ (UK Government, 2020b). It was not until 12 March – the day after the World Health Organization (WHO) officially declared Covid a pandemic – that the government announced that it was shifting to the ‘Delay’ phase of its strategy. Even then, the measures introduced were very limited – just advisory self-isolation for anyone displaying symptoms that might be attributable to Covid – with further measures, notably ‘social distancing measures for older and vulnerable people, asking them to self-isolate regardless of symptoms’, only to be introduced at some unspecified point ‘[i]n the coming weeks’. For the time being, the policy priority was to hold back, rather than to rush into action: ‘If we introduce this next stage too early, the measures will not protect us at the time of greatest risk but could have a huge social impact. We need to time this properly, continue to do the right thing at the right time, so we get the maximum effect for delaying the virus. … Our decisions are based on careful modelling’ (UK Government, 2020e).

Policymakers could invoke a wealth of modelling work to justify their decisions – including their decision to defer taking action. With the incorporation of SPI-M into SAGE, attention turned to modelling the effects of so-called ‘non-pharmaceutical interventions’ (NPIs), such as school closures, on the spread of the virus within the UK, particularly from late February, when SPI-M was asked to produce ‘a narrative describing effects of [NPIs] attempted in other countries, and develop illustrative scenarios showing the plausible impacts of combinations of interventions in the UK (simple visuals of epidemic curves)’ (SAGE, 2020c). The requested paper was ready for discussion at the SAGE meeting of 3 March, and noted among its findings that ‘The timing of the interventions would be critical’ (SAGE, 2020d). SAGE duly minuted that ‘Going forward, agreement on the optimal timing of these interventions will be required’, and tasked SPI-M with providing timings for discussion at the next meeting two days later (SAGE, 2020d). In the SAGE meetings on 5 March and 10 March, some dates and trigger points are suggested for specific interventions, including social distancing for 70+ and vulnerable groups (SAGE, 2020e, 2020f).

SPI-M’s stress on ‘timing’ followed from key assumptions incorporated into their modelling efforts. As requested by SAGE, SPI-M modellers aimed to determine how different interventions might affect the trajectory of an epidemic within the UK by flattening the epidemic curve, delaying and lowering the peak, and thereby reducing the total number of deaths that could be expected over the course of the epidemic. Crucially, the modellers assumed that it would be best to allow the epidemic to run its course in a single wave: excessive suppression of transmission was to be avoided since it would only lead to a second wave once the interventions were relaxed, thereby deferring but doing nothing to reduce the burden of illness and death. Their work therefore focused on fine-tuning the introduction of different interventions so as to maintain a level of infection calculated to minimize the overall death rate (SAGE, 2020e, 2020f). It was this work that the government invoked when arguing the need to defer introducing new measures until ‘the right time’.

Press coverage during the period from 1 to 12 March was largely uncritical both of government policy and of the rationale provided for it. Questions of timing had already begun to surface in the press by late February, notably in a *Guardian* editorial of 25 February which opined that the spread of Covid in the UK was practically inevitable, but ‘buying time can save lives’ (GU005). By 11 March, the *Guardian* was providing readers with ‘A coronavirus glossary’, explaining terms from the Action Plan including ‘Containment’, ‘Delay’ and ‘Flattened Curve’. The entry on ‘Delay’ read: ‘The UK is currently poised to move from a ‘containment’ to a ‘delay’ phase in managing the coronavirus outbreak. Delay reflects approaches to slow the spread of the disease if it is no longer possible to contain it – it is about buying time, both to ease pressure on health services, and to test possible drugs’ (GU007). On the same day, *The Guardian* also reported on a pre-print paper by modellers in Southampton, based on data from China, which likewise emphasized the importance of timing the introduction of NPIs (GU008).

That is not to say that the press paid no attention to the possible limitations of basing policy on models. *The Guardian* article just mentioned, for instance, included comments from two Edinburgh modellers, Mark Woolhouse and Rowland Kao, who pointed to the gap between scientific findings and policy making. Woolhouse was quoted as saying, ‘What is less clear from this analysis is what should happen next’, while Kao questioned how far models based on Chinese data could be applied to the UK (GU008). Despite such caveats, the piece was consistent with other reports in suggesting that, whatever the limitations of modelling, it provided the best guide to planning the policy response to Covid. A report on 9 March in the Scottish tabloid *The Daily Record* is typical in noting both the drawbacks of behavioural interventions and the importance of getting the timing right. Anticipating the introduction of ‘strict new measures … within the next 2 weeks’, the *Record* warned that the policy of self-isolation for anyone with possible Covid symptoms could ‘lead to millions being off work with minor colds and sniffles’. Nonetheless, the article as a whole generally endorsed the need to introduce such measures when the time was right, quoting extensively from the Prime Minister and his medical and scientific advisors, who in turn attributed their decisions to advice from modellers (DR001, MI001).

Indeed, this was the predominant tone of reporting on Covid policy and the role of modelling during this period (GU009). So long as the press generally approved of or at least acquiesced to the general speed and direction of policy, they were prepared to frame modelling as an uncertain but nonetheless invaluable basis for that policy. In particular, modelling was presented as providing a scientific basis from which to think about the temporal dynamics of the impending Covid epidemic and a rationale for not rushing into possibly damaging policy interventions. It was less clear that modelling could provide definitive guidance on exactly when such interventions should be implemented. In the absence of any urgent need to make a decision about timing, however, models continued to be accepted and endorsed as a justification for inaction. This acceptance would not last. Indeed, an official paper entitled ‘SPI-B insights on combined behavioural and social interventions’ dated 4 March 2020 presciently anticipates its failure:

Expectations of how the Government will react will be set by media reports of public health strategies in other countries. This increases the risk of public concern if interventions that are perceived to be effective are not applied. A clear explanation as to why expected interventions are not being implemented may be necessary. (UK Government, 2020d).

### Crisis: 13–16 March

Far from satisfying critics that the government had an effective policy for dealing with the Covid pandemic, the press conference of 12 March – and the announced shift from the ‘Contain’ to the ‘Delay’ phase of the government’s mitigation strategy – seems, if anything, to have intensified the press’s ambivalence about that strategy. In the days that followed, the press increasingly voiced doubts about government policy. Strains and tensions were now evident in the press framing of the Covid pandemic and how best to respond to it, with a notable loss of congruity between that framing and the one still adhered to by government policy makers and their advisors on SAGE.

Looming over the press’s reporting of Covid policy in this period, and a key element in the way the press framed that policy, was continuing concern that the UK government was taking a very different approach than were other national governments. Notably, on 22 February Italian policymakers had imposed a strict lockdown on a cluster of cities in the Lombardy and Veneto regions. On 8 March, with distressing images of hospitals in turmoil circulating widely, the order had been expanded to 15 northern regions, and escalated on 9 March to the first-ever nationwide lockdown imposed to curb the spread of a pandemic (Caduff, 2020; Ren, 2020). ‘Opposition parties have demanded to know why Britain is out of line with nations in which schools have been closed and cities put into lockdown’, reported *The Times* on 14 March, quoting the Plaid Cymru leader Liz Saville Roberts: “Government has a duty to answer questions: first and foremost, why take a different approach to other countries across the world?” (TI003). Scientists, too, were reported to be asking similar questions: ‘Many scientists said they were surprised that Britain had not imposed similar measures to those brought in by countries such as Italy, France, Ireland and China,’ noted *The Telegraph* (TE003). Philip Ball for *The Guardian* took the view that ‘There are too many unknowns to make reliable predictions, but Hong Kong and Singapore should be Britain’s role models,’ citing revered modeller, Roy Anderson, with a damning verdict on his own craft: ‘We just don’t know what the best strategy is. There are too many unknowns for mathematical models and predictions to be reliable’ (GU010).

The government responded by doubling down on its insistence that it was ‘following the science’, even going so far as to suggest that other countries were following other imperatives. But for many in the press, it was not just the policy, but also the science, that they now viewed with scepticism. For some, this meant accepting the strategy overall, but questioning whether modellers and policy makers had got the timing right. For instance, an op-ed by Juliet Samuel in *The Telegraph* on 13 March accepted that ‘The management of [the epidemic] … is all about timing’, but then struck a cautionary note. Observing that ‘The British gamble seems to be that the speed [of growth of the epidemic] can be fine-tuned’, Samuel warned that ‘If [the Government’s] modelling assumptions about the virus’s characteristics, the behaviour of the population or the capacity of the health service start to look dodgy, it needs to change tack at lightning speed’ (TE004). The following day, *The Times* reported former health secretary Jeremy Hunt’s admonition that ‘“the clock is ticking” to stop Britain becoming Italy’, and his concern that ‘The government is rightly following scientific advice – but even scientists have to make judgments and assumptions to inform their modelling’ (TI003). *The Telegraph* went further, noting that ‘The Government’s strategy of waiting until coronavirus is peaking before closing schools and asking vulnerable people to stay at home has been criticized by experts, who warned that further measures were needed to stop the epidemic spiralling out of control’ (TE003).

Other commentators began to question the entire strategy of attempting to manage the epidemic by timing the introduction of interventions, and pointed out that by acting promptly, other countries had succeeded in largely containing the virus. *The Guardian* was particularly vocal in expressing this view. Sarah Boseley noted that ‘Public health experts and hundreds of doctors and scientists at home and abroad are urging the UK government to change its strategy against coronavirus, amid fears it will mean the epidemic ‘lets rip’ through the population. They say the UK is turning its back on strategies that have successfully brought down the numbers of infections and deaths in other countries’ (GU011). Other *Guardian* writers cast their net more widely, citing an ongoing collapse of political and public support for government policy, and suggesting that those modellers and other experts who continued to support the policy of delay ‘expose an alarming schism in the nation’s attitudes to Covid and the attempts to control it’ (GU012).

Doubts about strategy extended to scepticism about the science on which it was supposedly based, with the press hosting a chorus of voices calling for the government’s models and data to be published so that it could be subjected to wider scrutiny. On 14 March, *The Times* published a letter from a group of public health experts led by Richard Horton (editor of *The Lancet*) urging ‘that the government urgently and openly share the scientific evidence, data and models it is using to inform its decisions on the Covid public health interventions in the UK. This transparency is essential to retain the scientific community, healthcare community, and the public’s understanding, co-operation and trust’ (TI004). An article by *The Times*’s health editor Chris Smyth amplified the message by quoting additional remarks by Horton to the effect that the policymakers were being downright disingenuous in claiming that their approach was science-led: ‘The UK government – [Health Secretary] Matt Hancock and [Prime Minister] Boris Johnson – claim they are following the science. That is not true. The evidence is clear.’ Smyth also quoted Jonathan Ashworth, the shadow health secretary: ‘I’m urging the government to offer greater clarity and publish more science so it can be better understood more widely, and peer-reviewed by experts in the field who are currently questioning our approach.’ Smyth even went so far as to express his own doubts about how policy makers were using science. Reporting Vallance’s view that ‘locking Britain down would result in more deaths than allowing a gradual peak in cases because the virus would bounce back later in the year’, he now added his own caveat: ‘However, the government has yet to publish the detailed figures behind its reasoning’ (TI003). *The Guardian* too was on the case, publishing a rash of readers’ letters on 13 March calling for the science to be published, and urging that public confidence would not be restored without greater transparency (GU013).

Mistrust in the scientific basis of government policy was only exacerbated when the Prime Minister and his advisers invoked the concept of herd immunity. On 13 March *The Telegraph* reported that ‘experts’ were warning that aiming for herd immunity was ‘a dangerous strategy, given that it was still unclear how the virus affected people and what the long-term implications were’ (TE003). The following day, *The Times* reported the views of Martin Hibberd, professor of emerging infectious disease at the London School of Hygiene & Tropical Medicine, who ‘expressed concerns about government planning that … aims for a controlled peak in the summer that gives the population herd immunity that protects the elderly. “I do worry that making plans that assume such a large proportion of the population will become infected (and hopefully recovered and immune) may not be the very best that we can do”’ (TI003). Also on 14 March *The Telegraph* reported that ‘not everyone agrees with herd immunity’, listing WHO spokesperson Margaret Harris and paediatrician Anthony Costello among the alleged dissenters (TE005). The following day, *The Guardian* published an article by American infectious disease epidemiologist William Hanage, who confided: ‘My colleagues here in the US … assumed that reports of the UK policy were satire – an example of the wry humour for which the country is famed’ (GU014).

Efforts by policymakers to row back on the idea that herd immunity was central to government strategy only partially allayed this scepticism. On 16 March, in an editorial on the topic, *The Times* pointed out that ‘A widespread belief that “herd immunity” was the strategy sprang directly from Boris Johnson’s own remarks on Friday, and those of Sir Patrick Vallance, the government’s chief scientific adviser … elucidating broad concern at an approach that appeared to differ markedly from that of every other nation.’ While the Health Secretary Matt Hancock subsequently ‘insisted that “herd immunity” …. was a scientific concept not a goal or a strategy’, *The Times* was only partly reassured: ‘This new message is a relief, but suggests a lack of coherence’ (TI005). In the same edition, *The Times* also reported behavioural scientist Susan Michie’s view that ‘public anxiety had been heightened by Britain seeming to take weaker measures than other countries and by confusion over herd immunity. She said that Mr Hancock should stop making announcements and the government’s two most prominent advisers must show that they had learnt from a series of setbacks’ (TI006). In *The Guardian*’s view, meanwhile, the herd immunity debacle had heightened the ‘sense of foreboding that the government is coming up short with its answers. … It was troubling – and reflects badly on ministers – that an epidemiological outcome of mass infection [i.e. herd immunity] was confused with the dubious policy aim of building resistance in the population’ (GU015).

The issue of herd immunity brought to a head the tensions in different framings of the Covid epidemic. Until the 12 March press conference, the press had largely acquiesced to the government’s policy decisions and the science that informed them. Accordingly, they had framed the British epidemic just as the *Daily Mail* continued to do: as a serious threat that could nonetheless be managed if the public trusted the government and their scientific advisers, who had the best available grasp of how the epidemic would develop and when to introduce more stringent preventive measures (DM001, DM002). After 12 March, however, many in the press inclined towards another framing, in which the threat of the disease was far more imminent, the appropriate policy response was exemplified by the emergency measures undertaken by other countries, and the scientific arguments put forward by the UK government’s policymakers and their advisers to justify their own policies were suspect – if not simply wrong. Moreover, where the former press framing was broadly congruent with that favoured by the government’s modellers and policymakers, the latter was distinctly at odds with the government’s framing. In effect, the days after the 12 March press conference were marked by an intensifying crisis of incongruency both with the press’s own framing of the epidemic and between the press and policy frames.

Meanwhile, behind the scenes, SAGE was also experiencing a crisis of its own, as urgent questions were raised in the SPI-M sub-committee about the adequacy of the current models/policy frame to address the growth of the epidemic in the UK. On 10 March, infectious disease epidemiologist Steven Riley circulated a short paper to his SPI-M colleagues, setting out his findings from a relatively simple model of the current epidemic. He pointed out, among other things, that the current policy of trying to manage the epidemic in a single wave would lead to critical care services being overwhelmed, and consequently to a death toll several times higher than expected under the current RWC scenario. Moreover, this would likely lead to affected populations spontaneously restricting their social contacts and interactions, much as would occur if the government took the lead in introducing lockdown measures. In consequence, Riley observed, the current government policy of attempting to manage the epidemic offered the worst of both worlds: On the one hand, it would entail a horrifically high death rate while still failing to achieve herd immunity; on the other, it would fail to avoid the economic consequence of lockdown. It would be far preferable, he argued, to adopt stringent but fixed-term measures to reduce current rates of infection, while using the time to develop alternative strategies (SAGE & Riley, 2020). Riley’s paper thus challenged the entire basis on which SPI-M, SAGE and the government were framing the UK’s Covid outbreak, and called for a major rethink of that frame (Farrar & Ahuja, 2021).

Over the following week, while SAGE members continued to try to explain and justify existing policy to the media, SPI-M members – particularly the Imperial, Cambridge and LSHTM teams – conducted further modelling in light of Riley’s intervention. While differing on the precise numbers, their findings confirmed the overall thrust and implications of Riley’s argument (Imperial College, Cambridge University, & SAGE, 2020; Imperial College & SAGE 2020; LSHTM & SAGE, 2020). By the time SAGE next met on 16 March, SPI-M as a whole was sufficiently persuaded of the merits of Riley’s case to submit a ‘consensus view’ on behavioural and social interventions, declaring ‘that a combination of case isolation, household isolation and social distancing of vulnerable groups is very unlikely to prevent critical care facilities being overwhelmed … [but] the addition of both general social distancing and school closures to case isolation, household isolation and social distancing of vulnerable groups would be likely to control the epidemic when kept in place for a long period. SPI-M-O agreed that this strategy should be followed as soon as practical, at least in the first instance’ (SPI-M-O, 2020). At that meeting, SAGE accordingly changed the advice it was offering to government: ‘1. On the basis of accumulating data, including on NHS critical care capacity, the advice from SAGE has changed regarding the speed of implementation of additional interventions. 2. SAGE advises that there is clear evidence to support additional social distancing measures be introduced as soon as possible’ (SAGE, 2020g).

Government responded with a flurry of new policy measures over the following week. On 16 March the Health Secretary Matt Hancock made a statement in the House of Commons advising the public to adopt more stringent social distancing measures, particularly if they were experiencing symptoms that could be attributed to Covid (Hancock, 2020). On the same day, the Prime Minister began holding daily briefings which he would use to announce a rapid ramping up of interventions: school closures on 18 March (Johnson, 2020b); closure of hospitality and entertainment venues on 20 March (Johnson, 2020); and compulsory stay-at-home measures – what would quickly come to be known as ‘lockdown’ – on 23 March (Johnson, 2020d). The new measures were summed up in the mantra ‘Stay at home. Protect the NHS. Save lives.’

In effect, this period saw a dramatic revision of the science/policy frame. Up to 16 March, the Covid problem had been framed in terms of a single epidemic curve that could be managed in such a way as to minimize the total number of deaths over the duration of the epidemic. From 16 March onwards, by contrast, the frame shifted to emphasize the high likelihood that, in the absence of stringent measures to restrict the spread of the virus, critical care facilities would be overwhelmed, with large numbers of deaths ensuing. Within that frame, strict lockdown now became the recommended appropriate solution.

Importantly for subsequent developments, that revision of the science/policy frame, and particularly the remedies that it now proposed, also served to resolve the tensions that had come to characterize the press frame over the preceding week. As we have seen, the press’s earlier acquiescence to the policy of ‘flattening the curve’ through carefully timed interventions had increasingly been challenged by an alternative framing in which immediate action was needed to avoid what was happening in Italy. The shift in government policy effectively brought it into alignment with this latter frame. With the escalation of policy interventions culminating in full lockdown, the press no longer needed to ask why the UK was not following Italian precedents. On the contrary, it was now able once again to speak in generally positive terms about government efforts to address the pandemic. This in turn had consequences for the role accorded to science, and especially modelling, within the press framing. More than simply being rehabilitated as a reliable guide to policy, modelling – and equally importantly, modellers – were now given even greater prominence in the press than they had enjoyed previously. From being little more than a bit player in the unfolding drama of Covid, modelling had now become a major player with a story all of its own.

### Domestication 16–30 March

The Prime Minister announced his change of policy at 5 pm on 16 March to the journalists who had gathered in Downing Street for the first of the new daily press conferences. Following the speech, the journalists put questions to the PM and his Chief Medical and Scientific Advisors. Two miles away, in the briefing room of the Science Media Centre on Euston Road, a second group of journalists watched a live stream of the Prime Minister’s statement (Science Media Centre, 2020). In the latter case, however, the statement was followed by face-to-face presentations from Neil Ferguson and Azra Ghani, two of the lead authors of the Imperial College modelling paper which had been discussed by SAGE earlier that day, informing SAGE’s revised advice to the government and ultimately leading to the change in policy that the PM had just announced. The paper itself was made public on the Imperial College website at the same time (Ferguson et al., 2020).

The Prime Minister made no mention of the Imperial College work in his speech, saying only that ‘according to SAGE it looks as though we’re now approaching the fast growth part of the upward curve’ (Johnson, 2020a). But journalists immediately drew the connection for themselves. Press reports over the following days, from broadsheets to tabloids, were practically unanimous in attributing the government’s change in policy to the work of the Imperial College modellers, with only *The Guardian* recognizing that ‘similar modelling from the London School of Hygiene and Tropical Medicine’ had also informed that decision (GU016, GU018). The shift in policy, as these reports put it, was variously ‘based on’ (GU016) or ‘resulted from’ (GU018) the modellers’ new findings, while modelling ‘was behind the government’s strategy change’ (GU017). The *Daily Mail* adopted more sensational language, declaring that the new modelling predictions had ‘forced the Government to U-turn dramatically’ (DM003). Many reports also mentioned Ferguson by name, and some singled him out as the principle agent of change. For *The Telegraph*, Ferguson was ‘the academic on whose data the decision was based’, who had seen that ‘[T]he change in strategy had been necessitated by new data … [and] had advised the government to change course’ (TE006). By 25 March, *The Telegraph* was referencing Ferguson as the ‘author of Government modelling on the pandemic’ (TE010).

This attribution of decisive agency to modellers sat uncomfortably with the way the Government sought to portray the policy process. The Prime Minister and his key advisors consistently spoke as if the new measures adopted from the 16 March onwards were simply a continuation of the strategy they had been following all along. Paul Nuki of *The Telegraph* wrote on 17 March that ‘The government is being careful not to say explicitly that it has switched course. … The chief scientific officer, Sir Patrick Vallance, said yesterday the difference was only ‘semantic’ …. It is difficult to read the modelling report and come to the same conclusion’, he commented (TE006).

Nuki was not alone in seeing a radical discontinuity where Vallance and Johnson sought to portray government policy as unaltered. The *Daily Mail* opined that the Imperial College team’s new ‘computer simulation’ had decisively demonstrated the unworkability of the government’s earlier strategy, based as it was on ‘the theory … that so-called herd immunity would kick in and the epidemic would die out’ (DM002). For *The Guardian*, writing with the clarity of hindsight, the Imperial College report had revealed a troubling obtuseness on the part of the Government: ‘It was clear that if Britain could not slow down the contagion, the disease would overload hospitals and kill hundreds in a few weeks, as it has done in northern Italy. That Boris Johnson did not fathom the depth of the danger is worrying. … What was obvious in Lombardy only became clear to Downing Street in the form of an analysis by epidemiologists at Imperial College London’ (GU019). Far from endorsing the government’s claim to have been ‘following the science’ all along, the press was now more inclined to portray policy makers as reluctant to abandon a discredited policy until forced to do so by Ferguson and his fellow modellers.

In effect, the press’s framing of the Covid problem had undergone a rapid reorientation. In the days before 16 March, as we have seen, the press was increasingly inclined to ask why Britain was not following Italy into lockdown, and increasingly mistrustful of both UK government policy makers and the modellers who advised them. After 16 March, when modelling advice was seen to shift decisively in favour of lockdown – thereby aligning with what the press had already been inclined to present as the necessary response to the escalating infection rate – the press now reframed modellers as authoritative advisers, responsible for forcing a reluctant policy establishment to make hard but necessary decisions. The fact that the same modellers had previously backed the now-discredited herd immunity strategy was no longer mentioned. Rather, the modellers’ own claims to have rapidly changed their advice in the light of ‘new information on the high rate of patients needing critical care in Italy’ (GU018) were taken at face value, as an indication of their ability to stay abreast of a rapidly changing situation (TE006, TE007).

The modellers themselves accordingly became figures of press interest. Ferguson, in particular, was hailed as ‘that valuable thing, a good scientist who is also a good communicator’, as well as a tireless ‘workaholic’ who continued to work even when he himself contracted coronavirus (GU018). But others too acquired name recognition, notably LSHTM modeller Adam Kucharski, whose book popularizing epidemiological science, fortuitously published in February, was now given glowing reviews in papers from the *Guardian* to the *Daily Mail* (GU020, DM004, Kucharski, 2020).

Not everyone in the press agreed with this heroic depiction of the modellers’ timely intervention in policy. *The Sun* continued to attribute agency to the policy makers, echoing the Prime Minister’s insistence that ‘he had to take “drastic” action to stop hospitals from being overwhelmed because the virus was spreading far faster than feared’, while portraying ‘the PM’s experts’ as merely ‘admit[ting] the coronavirus contagion is outstripping their modelling’ (SU001). In *The Telegraph*, contrarian columnist Sherelle Jacobs took an alternative view, attributing the shift in policy to the modellers, but not in a good way: The government should have stuck to its ‘creepy, clinical and completely correct’ herd immunity strategy to protect the economy, she argued, but ‘lost its nerve’ and ‘blinked, ditching herd immunity for an Imperial College research paper, which … preposterously argued that lockdown may have to continue for as long as 18 months, until a vaccine is found. This despite the fact there is no scientific consensus (a rival paper claims a few weeks of lockdown may be sufficient)’ (TE011). Others, better informed about the details of the case, argued that the modellers should have realized from the beginning that strict measures were needed to control the spread of the virus. Richard Horton, writing in *The Guardian*, rejected the modellers’ claims that ‘the science had changed’ and argued instead: ‘The science has been the same since January. What changed is that government advisers at last understood what had really taken place in China’ (GU021, GU022, TE012). Devi Sridhar concurred: ‘The earlier model did not include the ICU data shared in the Lancet on 24 January. Instead, it was similar, but much later information from Italy, that changed their recommendation’, (GU023), a sentiment shared by Nuki at *The Telegraph* (TE009).

The key point to note in all this, however, is that even those commentators who criticized the modellers or their impact on policy generally agreed (with the exception of *The Sun*) that they had played a crucial role in changing Covid policy. Moreover, by far the dominant framing in the press was that this was a good thing: Modellers had shown superior insight into the Covid problem and how to respond to it, and policy makers had been right to heed their advice. This carried over into continuing press interest in modelling and modellers through the remaining weeks of March. Not only did modelling feature more frequently in the newspapers than before the middle of the month; so too did a much wider range of modellers, as reporters looked beyond SAGE and SPI-M for findings and opinions to present to their readers.^3^ Modelling now came to be seen as a reliable means of providing evidence and insight into the pros and cons of a range of possible interventions.

With this broadening perspective, the press also started to present a more nuanced view of the uncertainties and limitations surrounding modelling and the predictions and prognostications it offered to policy makers. This was exemplified in the cautious reporting of findings from a team of Oxford-based modellers, who appeared to suggest that a much larger proportion of the UK population than indicated by previous models might already have had the virus and acquired immunity (DR002, GU026).

Finally, with modelling now central to the way that the press framed the Covid problem, ideas and language derived from epidemiological modelling became part of the way that the press itself talked about the epidemic, for instance when considering whether lockdown would ‘achieve its aim of flattening the curve and cutting the death toll’ (HE002). At the same time, policymakers’ use of the same language now began once again to be uncritically reproduced in press reports, as for instance when *The Telegraph* quoted Deputy CMO Jenny Harries’s claim that ‘we are watching the curve …. The issue here is, exactly as we have done all the way through, about keeping watching the epidemiology and flexing those interventions at the right time, in the right place, to deliver what we need’ (TE013). *The Guardian* likewise saw no need to qualify or question Michael Gove’s statement that ‘We aim to flatten the curve, reduce the rate of infection to ensure the NHS can be protected’ (GU026). In effect, government policy makers’ continuing use of the now authoritative and trusted language of infectious disease modelling served to reinstate them in the press’s framing of the COVID crisis, enabling them to claim a continuity of policy that the press had previously doubted. *The Guardian*, for instance, quoted Harries as saying that ‘the government has always acted on the science in this unprecedented event. Data changes frequently, so there is a moving agenda on some of the data, but be clear that we have acted on modelling and steadily implemented measures in a way that they are timed appropriately’ (GU026). Policymakers were once more able to claim to be working in partnership with modellers in a way that was responsive to rapidly changing circumstances. As NHS England Medical Director Stephen Powis put it: ‘[T]he scientists who are working with the Government to model what we can expect are adjusting their predictions now as we start to see the actuality of the epidemic in the UK, rather than what we believed might have happened a few weeks ago’ (DR003). By mobilizing the language of modelling in this way, policymakers now represented themselves not simply as following the science, but actively working with modellers to stay on top of a rapidly changing situation.

## Discussion and conclusion

Domesticated modelling, the pervasive perception and application of modelling as leading expertise in pandemic policy, is not solely an effect of the science’s predictive capacity. Rather, the assent that models came to command depended on the remarkable ‘interpretative flexibility’ that they exhibited between early January and late March 2020. This period, as we show above, was marked neither by a steady increase of trust in inferences from modelling, nor by any consistency of recommendations provided by either modellers or by policymakers in the UK. Rather, if we consider the significant variability, and at times contrariness, with which the UK press framed the modelling-policy nexus, the important question becomes why and how modelling came to be central to the story of the UK response to Covid in spite of a very public crisis of credibility.

We show that the first period was shaped by contingency, where the characteristic uncertainty of modelling coalesced with doubts about the likelihood of Covid ever even taking hold in the UK. Modelling was framed in this period as a practice of speculation, neither equipped with authority, nor presumed to offer reliable inferences. The second period was structured by considerations of timing, with modelling framed as a practice of prevarication. In this period, models were tasked with projecting increasing numbers, predicting peaks and, crucially, justifying government inaction. Over the third period, as public anxiety over that inaction mounted, modelling suffered a credibility crisis. Framed as unreliable, risky, and reckless, the field stood accused of being complicit in the government’s apparent failure to react appropriately to the emerging epidemic. This crisis was resolved when key models were dramatically revised to call for urgent action to limit the spread of the epidemic and – equally dramatically – the government announced the introduction of lockdown. In the fourth and final period, with modelling now back in line with what was widely presumed to be an appropriate response to the pandemic, modelling was again reframed as both the justification and the necessary stimulus for government policy.

Our analysis temporalizes the conditions under which modelling became domesticated as a trusted and reliable guide in a pandemic crisis. The apparent ability of models to compel government action, command wide popular assent, and define the contours of the pandemic was preceded by periods of speculation, prolonged prevarication and a substantial crisis of credibility. In those periods, while models certainly became part of ‘evidence-making assemblages’, they did not act as ‘performative actors’ at the level of public health policy (Rhodes & Lancaster, 2020, 2021). With the Ferguson paper, modelling might have begun to define the contours of the ‘crisis’ (Anderson, 2021, p. 171), but this occurred only as a consequence of the alignment of projections derived from modelling with wider expectations of what modelling should demonstrate. As modellers revised their models in ways that acknowledged what was seen to be happening in China and Italy, and as government adopted a more interventionist approach to policy, so the press reframed modelling as an objective and reliable guide to action. Under these ‘conditions of felicity’ (MacKenzie, 2006, p. 43), infectious disease modelling was finally enabled to define the shape of the pandemic. Invested with the authority to shift government policy, models were published and discussed in the press, and the language of modelling became domesticated as pandemic vernacular.

Our epistemological microhistory of Covid modelling in the UK shows that large parts of the series of events that led to lockdown and the subsequent domestication of modelling stand in stark contrast to the image of pervasive influence stemming from infectious disease modelling. Our narrative of speculation, prevarication, crisis and resolution in domestication suggests rather that modelling came to prominence in the press – indeed came to be elevated as the basis of sound policymaking – because it was implicated in a change of policy that resolved an immediate anxiety about why the UK was not following the example of other countries. Perhaps most surprisingly, our findings suggest that modelling gained exposure and credibility because of the role it was seen to play in achieving a short-term fix, not due to the science’s presumed capacity for projecting long-term options.

Why does this nuancing of how models became used and trusted matter? That a scientific practice rises to a challenge in a non-linear fashion, and that the authority of scientific knowledge claims is not independent of political and societal influences, is hardly a novel revelation. But our analysis of the temporal patterning of domestication also captures how the acceptance of modelling extends beyond the instruction of policy action. The same re-framing that represented models as the drivers of a dramatic policy U-turn also projected an imagined continuity of the modelling-led-policy frame into the past. In the domestication period, as reporting on modelling and policy finally assumed congruency, previous periods of speculation, prevarication and crisis disappeared almost immediately from the press and the records of writers, scientists, and policymakers alike.

As truth devices, it is now widely supposed that models have consistently informed and guided the UK government’s Covid policy, not just in the present but in the periods preceding the lockdown. We may refer to this as the historiality of models – a particular instance of the ‘historial structure of scientific action’ (Rheinberger, 1999, p. 67). Models were retrospectively invested with the inherent power to compel policy into action, apparently independently of external conditions. As recursive constructs, models make history as much as they inform the present and project the future.

The media analysis we have advanced offers an empirical revision of conceptual approaches to understanding the role of models in the response to Covid by foregrounding the significance of temporal analysis. With much of the literature in the social studies of Covid models concerned with the material, ontological or mathematical power of this scientific craft, our analysis returns to the conditions under which models become invested with authority *over time* and *in between* policy, the media and the public. Without such temporal sensitivity, the study of modelling in and beyond Covid risks reinstating narratives of the inherent quality of models to compel action, which we instead identify as a contingent and recurrent effect of models’ performances.

## Supplemental Material

sj-docx-1-sss-10.1177_03063127221126166 – Supplemental material for Domesticating models: On the contingency of Covid-19 modelling in UK media and policy Click here for additional data file. Supplemental material, sj-docx-1-sss-10.1177_03063127221126166 for Domesticating models: On the contingency of Covid-19 modelling in UK media and policy by Lukas Engelmann, Catherine M Montgomery, Steve Sturdy and Cristina Moreno Lozano in Social Studies of Science

## Notes

1. See Supplemental Appendix 1 for bibliographic data on cited newspaper articles.

2. As of 26 February, the RWC included an infection attack rate (IAR) of 80% of the population and an infection mortality rate (IMR) of 1%, but listed excess deaths as ‘Unknown, use pan flu planning assumptions’ (UK Government, 2020a). In the updated RWC of 3 March, the estimates for the IAR and IMR remained unchanged, but were now used to calculate an excess mortality figure of 520,000 (UK Government, 2020c). The reason for this change is not clear.

3. The modellers, broadly understood, who now featured in the press included Francois Balloux, professor of computational systems biology and the director of the University College London Genetics Institute (HE001); the Southampton WorldPop team inc. modelling the effectiveness of contact tracing (TE008); Cambridge modellers Sheila Bird and David Spiegelhalter (GU024); tumor modeller Inigo Martincorena (TE009); Dr Ari Ercole from division of anaesthesia, University of Cambridge modelling ICU demand (GU025).
