# Supplementary material for: Domesticating models: On the contingency of Covid-19 modelling in UK media and policy
Source: Soc Stud Sci. 2022 Oct 13;53(1):121–45. doi: 10.1177/03063127221126166 (PMC9892880; doi:10.1177/03063127221126166)
Supplement: sj-docx-1-sss-10.1177_03063127221126166 – Supplemental material for Domesticating models: On the contingency of Covid-19 modelling in UK media and policy [file sj-docx-1-sss-10.1177_03063127221126166.docx]

Appendix 1: Research Data

*Daily Record*

DR001: Hughes, David. ‘Anyone with Flu Symptoms to Be Told to Self-Isolate as UK Prepares for next Phase of Coronavirus - Daily Record’. *Daily Record*, 9 March 2020. <https://www.dailyrecord.co.uk/news/uk-world-news/anyone-flu-symptoms-told-self-21663039>.

DR002: Bagot, Martin. ‘Hope for Brits in UK Lockdown’. *Daily Record*, 26 March 2020.

DR003: Blackstock, Gordon. ‘BRACED FOR 1000 DEATHS;CORONACRISIS REFRIGERATED UNITS SET TO BE USED ; AS EMERGENCY MORGUES ACROSS SCOTLAND ; Army Steps in to Help Plan for Inevitable Rise in Covid-19 Fatalities as Chief Medical Officer Says 65,000 Scots Are Likely to Have the Virus’. *Daily Record*, 29 March 2020.

*The Daily Mail*

DM001: Borland, Sophie. ‘NHS in Highest Alert: Health Chiefs Say Coronavirus Is “Level Four Emergency”’. *Mail Online*, 3 March 2020, sec. News. <https://www.dailymail.co.uk/news/article-8071721/NHS-highest-alert-Health-chiefs-say-coronavirus-level-four-emergency.html>.

DM002: Elsom, Jack. ‘Coronavirus Deaths in the UK Almost Double Overnight to 21’. *Mail Online*, 14 March 2020, sec. News. <https://www.dailymail.co.uk/news/article-8112177/Coronavirus-deaths-UK-double-overnight-21.html>.

DM003: Naish, John. ‘The Dossier That Horrified British Experts’. *Mail Online*, 17 March 2020, sec. News. <https://www.dailymail.co.uk/news/article-8123677/The-dossier-horrified-British-experts.html>.

DM004: Alton, Roger. ‘Why Things Go VIRAL and Why the World Goes Mad When They Do’. *Mail Online*, 19 March 2020, sec. Books. <https://www.dailymail.co.uk/home/books/article-8132335/Why-things-VIRAL-world-goes-mad-do.html>.

*The Herald*

HE001: Glass, Jess. ‘“There Might Not Be an Acceptable Solution to the Problem”’. *The Herald*, 16 March 2020.

HE002: Scott, Garry. ‘Glimmers of Hope amid the Growing Fears over Covid-19’. *The Herald*, 26 March 2020.

*The Mirror*

MI001: Bagot, Martin, Pippa Crera, and Orlaith Clinton. ‘CORONAVIRUS CRISIS: TWO N.I. SCHOOLS SHUT Deep Clean after Pupil Tests Positive People with Flu Signs “to Isolate” 2 New Deaths as Outbreak Worsens’. *The Mirror*, 10 March 2020.

*The Guardian*

GU001: Hodal, Kate, Sarah Boseley, and Calla Wahlquist. ‘Coronavirus: More Cases and Second Death Reported in China’. *The Guardian*, 18 January 2020, sec. World news. <http://www.theguardian.com/world/2020/jan/17/corona-second-death-in-china-after-sars-like-outbreak>.

GU002: Boseley, Sarah. ‘Coronavirus: Heathrow to Screen Arrivals from Affected Chinese Region’. *The Guardian*, 22 January 2020, sec. World news. <http://www.theguardian.com/science/2020/jan/22/coronavirus-heathrow-to-create-separate-arrival-area>.

GU003: Boseley, Sarah, and Rowena Mason. ‘Coronavirus: 100,000 May Already Be Infected, Experts Warn’. *The Guardian*, 26 January 2020, sec. World news. <http://www.theguardian.com/science/2020/jan/26/coronavirus-could-infect-100000-globally-experts-warn>.

GU004: Editors. ‘The Guardian View on the New Coronavirus: Be Alert, Not Afraid | Editorial’. *The Guardian*, 21 January 2020, sec. Opinion. <http://www.theguardian.com/commentisfree/2020/jan/21/the-guardian-view-on-the-new-coronavirus-be-alert-not-afraid>.

GU005: Editors. ‘The Guardian View on the New Coronavirus: Buying Time Can Save Lives | Editorial’. *The Guardian*, 25 February 2020, sec. Opinion. <http://www.theguardian.com/commentisfree/2020/feb/25/the-guardian-view-on-the-new-coronavirus-buying-time-can-save-lives>.

GU006: Editors. ‘The Guardian View on China and the Coronavirus: Scrutiny, Not Stigma | Editorial’. *The Guardian*, 29 January 2020, sec. Opinion. <http://www.theguardian.com/commentisfree/2020/jan/29/the-guardian-view-on-china-and-the-coronavirus-scrutiny-not-stigma>.

GU007: Davis, Nicola. ‘From Ancestral Strain to Zoonosis: A Coronavirus Glossary’. *The Guardian*, 11 March 2020, sec. World news. <http://www.theguardian.com/world/2020/mar/11/from-ancestral-strain-to-zoonosis-a-coronavirus-glossary>.

GU008: Sample, Ian. ‘Research Finds Huge Impact of Interventions on Spread of Covid-19 | Coronavirus | The Guardian’. *The Guardian*, 11 March 2020. <https://www.theguardian.com/world/2020/mar/11/research-finds-huge-impact-of-interventions-on-spread-of-covid-19>.

GU009: Proctor, Kate. ‘UK Government’s Coronavirus Advice – and Why It Gave It’. *The Guardian*, 12 March 2020, sec. World news. <http://www.theguardian.com/world/2020/mar/12/uk-governments-coronavirus-advice-and-why-it-gave-it>.

GU010: Ball, Philip. ‘What Can Britain Learn about Containing Covid-19 from Countries That Got It Right?’ *The Guardian*, 12 March 2020, sec. Opinion. <http://www.theguardian.com/commentisfree/2020/mar/12/britain-containing-covid-19-countries-hong-kong-singapore>.

GU011: Boseley, Sarah. ‘Coronavirus: Health Experts Fear Epidemic Will “Let Rip” through UK’. *The Guardian*, 15 March 2020, sec. World news. <http://www.theguardian.com/world/2020/mar/15/coronavirus-health-experts-fear-epidemic-will-let-rip-through-uk>.

GU012: Helm, Toby, and Robin Mckie. ‘Coronavirus: How Johnson’s Plan Shifted as Virus Wreaked Havoc’. *The Guardian*, 15 March 2020, sec. World news. <http://www.theguardian.com/world/2020/mar/15/boris-johnson-coronavirus-outbreak-how-strategy-shifted>.

GU013: Letters. ‘This Government Must Show Us the Evidence behind Its Covid-19 Policies | Letters’. *The Guardian*, 13 March 2020, sec. World news. <http://www.theguardian.com/world/2020/mar/13/this-government-must-show-us-the-evidence-behind-its-covid-19-policies>.

GU014: Hanage, William P. ‘I’m an Epidemiologist. When I Heard about Britain’s “Herd Immunity” Coronavirus Plan, I Thought It Was Satire | William Hanage’. *The Guardian*, 15 March 2020, sec. Opinion. <http://www.theguardian.com/commentisfree/2020/mar/15/epidemiologist-britain-herd-immunity-coronavirus-covid-19>.

GU015: Editors. ‘The Guardian View on the UK’s Covid-19 Response: Confused and Hesitant | Editorial’. *The Guardian*, 15 March 2020, sec. Opinion. <http://www.theguardian.com/commentisfree/2020/mar/15/the-guardian-view-on-the-uks-covid-19-response-confused-and-hesitant>.

GU016: Stewart, Heather, Sarah Boseley, Peter Walker, and Larry Elliot. ‘PM Tells Britons to Avoid Non-Essential Travel and Contact | Coronavirus | The Guardian’. *The Guardian*, 16 March 2020. <https://www.theguardian.com/world/2020/mar/16/pm-tells-britons-to-avoid-non-essential-contact-with-others>.

GU017: Proctor, Kate. ‘Shutting Schools Would Reduce Coronavirus Transmission – Expert’. *The Guardian*, 17 March 2020, sec. Education. <http://www.theguardian.com/education/2020/mar/17/shutting-schools-would-reduce-coronavirus-transmission-expert>.

GU018: Boseley, Sarah. ‘Neil Ferguson: Coronavirus Expert Who Is Working on despite Symptoms’. *The Guardian*, 18 March 2020, sec. World news. <http://www.theguardian.com/world/2020/mar/18/neil-ferguson-coronavirus-expert-who-is-working-on-despite-symptoms>.

GU019: Editors. ‘The Guardian View on the UK’s Covid-19 Economic Plan: Fine Sentiment, but Lacks Details | Editorial’. *The Guardian*, 17 March 2020, sec. Opinion. <http://www.theguardian.com/commentisfree/2020/mar/17/the-guardian-view-on-the-uks-covid-19-economic-plan-fine-sentiment-but-lacks-details>.

GU020: Spinney, Laura. ‘The Rules of Contagion by Adam Kucharski Review – Outbreaks of All Kinds’. *The Guardian*, 25 March 2020, sec. Books. <http://www.theguardian.com/books/2020/mar/25/the-rules-of-contagion-by-adam-kucharski-review-outbreaks-of-all-kinds>.

GU021: Horton, Richard. ‘Scientists Have Been Sounding the Alarm on Coronavirus for Months. Why Did Britain Fail to Act?’ *The Guardian*, 18 March 2020, sec. Opinion. <http://www.theguardian.com/commentisfree/2020/mar/18/coronavirus-uk-expert-advice-wrong>.

GU022:Sample, Ian. ‘UK Failures over Covid-19 Will Increase Death Toll, Says Leading Doctor’. *The Guardian*, 18 March 2020, sec. World news. <http://www.theguardian.com/society/2020/mar/18/uk-failures-over-covid-19-will-increase-death-toll-says-leading-doctor>.

GU023: Sridhar, Devi. ‘Britain Had a Head Start on Covid-19, but Our Leaders Squandered It | Devi Sridhar’. *The Guardian*, 23 March 2020, sec. Opinion. <http://www.theguardian.com/commentisfree/2020/mar/23/britain-covid-19-head-start-squandered>.

GU024: Sample, Ian. ‘Coronavirus: How Do Italy and the UK Compare?’ *The Guardian*, 23 March 2020, sec. World news. <http://www.theguardian.com/world/2020/mar/23/coronavirus-how-do-italy-and-the-uk-compare>.

GU025: Boseley, Sarah. ‘NHS Hospitals Could Run out of Coronavirus Beds in a Fortnight’. *The Guardian*, 24 March 2020, sec. Society. <http://www.theguardian.com/society/2020/mar/24/nhs-hospitals-could-run-out-of-coronavirus-beds-in-a-fortnight>.

GU026: Costello, Anthony. ‘Mass Testing Is the Only Way to Stop the Virus – It’s Long Overdue’. *The Guardian*, 25 March 2020, sec. Opinion. <http://www.theguardian.com/commentisfree/2020/mar/25/mass-covid-19-testing-is-vital-but-the-data-must-be-localised>.

GU027: Campbell, Lucy, Amy Walker, Damien Gayle, and Alex Hern. ‘UK Coronavirus Live: Rate of Infection Doubling Every Three to Four Days, Says Gove – as It Happened’. *The Guardian*, 27 March 2020, sec. Politics. <https://www.theguardian.com/politics/live/2020/mar/27/uk-coronavirus-live-rough-sleepers-nhs-applause-covid-19-latest-news>.

*The Telegraph*

TE001: Gulland, Anne. ‘Death Rate for Patients Hospitalised with Coronavirus in Wuhan Nearly 20 per Cent, Study Shows’. *The Telegraph*, 10 February 2020. <https://www.telegraph.co.uk/global-health/science-and-disease/death-rate-coronavirus-wuhan-nearly-20-per-cent-study-shows/>.

TE002: Gulland, Anne. ‘Coronavirus May Be Spreading Undetected Outside China’. *The Telegraph*, 17 February 2020. <https://www.telegraph.co.uk/global-health/science-and-disease/coronavirus-may-spreading-undetected-outside-china/>.

TE003: Knapton, Sarah, and Gordon Rayner. ‘Government Strategy of Delaying Measures until Coronavirus Peaks Is Risky, Experts Warn’. *The Telegraph*, 13 March 2020. <https://www.telegraph.co.uk/politics/2020/03/13/government-following-dangerous-strategy-waiting-coronavirus/>.

TE004: Samuel, Juliet. ‘The Government Wants Us to Get Ill – but Not Too Ill’. *The Telegraph*, 13 March 2020. <https://www.telegraph.co.uk/news/2020/03/13/government-wants-us-get-not/>.

TE005: Davies, Gareth. ‘What Is Herd Immunity and Will It Stop Coronavirus in the UK?’ *The Telegraph*, 15 March 2020. <https://www.telegraph.co.uk/news/2020/03/15/what-herd-immunity-mean-will-stop-coronavirus-uk/>.

TE006: Nuki, Paul. ‘The Terrifying Data behind the Government’s Sudden Coronavirus Lockdown’. *The Telegraph*, 17 March 2020. <https://www.telegraph.co.uk/global-health/science-and-disease/terrifying-data-behind-government-coronavirus-lockdown/>.

TE007: Newey, Sarah. ‘Almost One in Three People given Hospital Treatment for Coronavirus Will Need Intensive Care’. 18 March 2020,

<https://www.telegraph.co.uk/global-health/science-and-disease/coronavirus-hospitalisation-rates-revealed-80-per-cent-infected/>.

TE008: Knapton, Sarah, and Henry Bodkin. ‘Coronavirus: The Unintended Consequences of the UK Lockdown and Why Millions of People Could Already Be Infected’. *The Telegraph*, 20 March 2020. <https://www.telegraph.co.uk/news/2020/03/20/coronavirus-unintended-consequences-uk-lockdown-millions-people/>.

TE009: Nuki, Paul. ‘We Are 14 Days behind Italy’s Coronavirus Crisis: When Will Britain Wake up to the Sobering Reality?’ *The Telegraph*, 22 March 2020. <https://www.telegraph.co.uk/global-health/science-and-disease/storm-coming-experts-say-14-days-behind-italy-rules-uk-bed-rationing/>.

TE010: Donnelly, Laura. ‘Coronavirus Home-Testing Kits Available to Public “within Weeks”’. *The Telegraph*, 25 March 2020. <https://www.telegraph.co.uk/news/2020/03/25/coronavirus-home-testing-kits-available-public-within-weeks/>.

TE011: Jacobs, Sherelle. ‘The PM Was Panicked into Abandoning a Sensible Covid-19 Strategy, and Has Plunged Society into Crisis’. *The Telegraph*, 26 March 2020. <https://www.telegraph.co.uk/politics/2020/03/26/lockdown-wests-berlin-wall-moment-elite-managerialism-collapses/>.

TE012: Knapton, Sarah. ‘Two Thirds of Coronavirus Victims May Have Died This Year Anyway, Government Adviser Says’. *The Telegraph*, 25 March 2020. <https://www.telegraph.co.uk/news/2020/03/25/two-thirds-patients-die-coronavirus-would-have-died-year-anyway/>.

TE013: Mendick, Robert, and Danielle Sheridan. ‘“More Flexible” Coronavirus Lockdown Could Last Six Months, Senior Government Health Adviser Warns’. *The Telegraph*, 26 March 2020. <https://www.telegraph.co.uk/news/2020/03/26/flexible-coronavirus-lockdown-could-last-six-months-senior-government/>.

*The Times*

TI001: Smyth, Chris. ‘First Coronavirus Death in Europe Reported in France’, 15 February 2020, sec. news. <https://www.thetimes.co.uk/article/coronavirus-half-of-britons-would-fall-ill-in-worst-case-outbreak-57g5qbqnw>.

TI002: Karim, Fariha, Didi Tang, and Emma Yeomans. ‘Coronavirus: We’ve Been Abandoned, Britons Stranded Aboard Cruise Ship Fear’, 17 February 2020, sec. news. <https://www.thetimes.co.uk/article/hundreds-of-american-passengers-evacuated-from-coronavirus-cruise-ship-diamond-princess-6mp8cvg8t>.

TI003: Smyth, Chris, Matt Chorley, and Katie Gibbons. ‘Coronavirus: Ministers Urged to Come Clean on Model for British Response’, 14 March 2020, sec. news. <https://www.thetimes.co.uk/article/coronavirus-ministers-urged-to-come-clean-on-model-for-british-response-qntbr3mn3>.

TI004: Horton, Richard. ‘Coronavirus Modelling “Must Be Made Clear”’, 14 March 2020, sec. comment. <https://www.thetimes.co.uk/article/coronavirus-modelling-must-be-made-clear-3zcl7x578>.

TI005: Editors. ‘The Times View on Britain’s Coronavirus Plans: Clear and Calm’, 16 March 2020, sec. comment. <https://www.thetimes.co.uk/article/the-times-view-on-coronavirus-plans-clear-and-calm-wlq9w9bpv>.

TI006: Smyth, Chris, Lucy Fisher, Kat Lay, and Greg Hurst. ‘Coronavirus: Schools Told to Isolate Pupils with Cough’, 16 March 2020, sec. news. <https://www.thetimes.co.uk/article/coronavirus-pandemic-pm-tries-to-regain-grip-as-death-toll-rises-to-35-6rg6nzzmw>.

*The Sun*

SU001: Newton Dunn, Tom. ‘AVOID PUBS, CLUBS AND PEOPLE; PM TO BRITS: END NON-ESSENTIAL CONTACT’. *The Sun*, 17 March 2020.
